# Optimizing wheat prosperity: innovative drip irrigation and nitrogen management strategies for enhanced yield and quality of winter wheat in the Huang-Huai-Hai region

**DOI:** 10.3389/fpls.2024.1454205

**Published:** 2024-08-30

**Authors:** Jinke Zhao, Xuexin Xu, Shuai Liu, Jing Jia, Menglan Li, Hanliu Huang, Guoqing Zhang, Changxing Zhao

**Affiliations:** Shandong Provincial Key Laboratory of Dryland Farming Technology, College of Agronomy, Qingdao Agricultural University, Qingdao, China

**Keywords:** winter wheat, drip irrigation, water and nitrogen combination, growth period, grain yield, grain quality

## Abstract

**Introduction:**

To examine the impacts of varied water and nitroge combinations on wheat yield and quality under drip irrigation in the Huang-Huai-Hai area, a field experiment was conducted over two growing seasons of winter wheat from 2019 to 2021.

**Methods:**

Traditional irrigation and fertilization methods served as the control (CK), with two nitrogen application rates set: N1 (180 kg/ha) and N2 (210 kg/ha). The irrigation schedules were differentiated by growth stages: jointing, anthesis (S2); jointing, anthesis, and filling (S3); and jointing, booting, anthesis, and filling (S4), at soil depths of 0-10 cm (M1) and 0-20 cm (M2).

**Results:**

Results indicated that compared to CK, the 3 and 4 times irrigation treatments comprehensively improved grain yield (GY) by 8.0% and 13.6% respectively, increased the average plant partial factor productivity of nitrogen fertilizer (PFPN) and irrigation use efficiency (IUE) by 57.5% and 38.2%, and 62.2% and 35.8%, respectively. The gluten content (GC) of 3 irrigations was 1.6% higher than CK, and other metrics such as dough tenacity (DT), softness (ST), water absorption (WAS), and gluten hardness (GH) also showed improvements. Furthermore, the contents of amylose, amylopectin, and total starch under 3 irrigations significantly increased by 9.4%, 11.4%, and 9.8%, respectively, with higher than 4 irrigations. The crude protein content and soluble sugar content in 3 irrigations rose by 6.5% and 9.8% respectively over two years. These irrigation treatments also optimized gelatinization characteristics of grains, such as breakdown viscosity (BDV), consistency peak viscosity (CPV), consistency setback viscosity (CSV), pasting temperature (PeT), and pasting time (PaT).

**Discussion:**

The study demonstrated that appropriate drip irrigation can effectively synchronize water and nitrogen supply during critical growth stages in winter wheat, ensuring robust late-stage development and efficient transfer of photosynthetic products into the grains, thus enhancing grain mass and yield. This also led to improved utilization of water and fertilizer and enhanced the nutritional and processing quality of the grain. However, excessive irrigation did not further improve grain quality. In conclusion, given the goals of saving water and fertilizer, achieving excellent yield, and ensuring high quality, the N1S3M1 treatment is recommended as an effective production management strategy in the Huang-Huai Hai area; N1S3M2 could be considered in years of water scarcity.

## 1 Introduction

Wheat is a primary source of protein and starch in our diet. The wheat output from the Huang-Huai-Hai area constitutes about 70% of the total wheat production in China (Qin et al., 2024). In this region, agricultural water scarcity, insufficient rainfall during the wheat growing season, and challenges with topdressing hinder wheat growth in its later stages, limiting high-quality wheat production (Ding et al., 2017). Local agronomic management practices, such as border irrigation with large amounts of fertilizer, exacerbate water shortages, waste resources, and cause environmental pollution (Hu et al., 2023). Given that the current wheat yield and quality do not meet the demands of daily life, the wheat production target in the Huang-Huai-Hai area has shifted from merely focusing on high and stable yield to improving both yield and quality (Ma et al., 2019). Therefore, exploring high-yield and high-quality dryland wheat cultivation technology is crucial for ensuring grain production security in the Huang-Huai-Hai area.

Reasonable cultivation measures are crucial for controlling the yield and quality of wheat grains (Olga et al., 2022). External nitrogen application effectively promotes nitrogen accumulation, yield, and quality in wheat. External nitrogen application effectively promotes nitrogen accumulation, yield, and quality in wheat (Olga et al., 2018). However, a sole focus on yield has led to indiscriminate nitrogen application, resulting in ineffective tillering, increased soil nitrogen residue, and reduced fertilizer efficiency and utilization rates (Zheng et al., 2020). The interaction between irrigation methods and nitrogen application significantly improves wheat yield and quality (Xin et al., 2023). Rational water and nitrogen management can markedly enhance wheat yield and fertilizer utilization efficiency. Rational water and nitrogen management can markedly enhance wheat yield and fertilizer utilization efficiency (Li et al., 2011). Compared to other irrigation methods, the integration of drip irrigation with water and fertilizer offers significant advantages in saving water and fertilizer, reducing labor, achieving high yield and efficiency, and plays a vital role in high-quality agricultural production and sustainable development in water-scarce regions (Cui et al., 2023; Yao et al., 2023a).

Starch is synthesized by the input of photocontract compounds into the grain as sucrose, catalyzed by a series of enzymes (Hu et al., 2023). Soluble sugar, the substrate for starch synthesis, is closely related to starch accumulation (Qu et al., 2018). Quality indicators such as grain hardness, settling value, wet gluten content, protein content, silty parameters, and tensile parameters are essential for determining wheat use (Ma et al., 2021; Peng et al., 2022). Various amounts and frequencies of water and fertilizer application significantly affect wheat quality and yield. Nitrogen application at the jointing stage promotes nitrogen transfer from vegetative organs to the grain, enhancing grain protein content and improving wheat processing quality. Appropriate nitrogen application significantly increases wheat grain yield, protein content, gluten content, precipitation value, dough stability time, and dough tension. However, excessive nitrogen application reduces grain yield, processing quality, and nutritional quality of wheat (Otteson et al., 2007; Zheng et al., 2018). Early-stage irrigation benefits grain protein accumulation, while reducing irrigation during the middle and late stages increases grain protein content and sedimentation value, prolonging dough formation and stability time (Li et al., 2021). A study for “Jimai 20” which a strong gluten wheat variety, irrigating at the post-anthesis stages significantly improved grain protein content and processing quality. Grain yield, dough formation time, and stability time of irrigation after anthesis were significantly higher than those of subsequent irrigations (Wang et al., 2024). For nitrogen application rates ranging from 0 to 210 kg/ha, wheat grain protein content, wet gluten content, gluten content, and settling value were negatively correlated with irrigation frequency under the same fertilizer application rate (Yao et al., 2023b). Additionally, under 180 kg/ha nitrogen application, GY and water absorption were positively correlated with irrigation frequency, while grain hardness, bulk density, dough stability time, and formation time were negatively correlated (Li et al., 2014). There were also studies that believe winter wheat in North China does not require irrigation in wet years, but irrigation of 60-75 mm at the jointing and heading stages in normal and dry years can significantly improve grain yield and quality (Wang et al., 2006).

Previous studies have yielded a series of results regarding the effects of different amounts of water and fertilizer application on wheat quality and yield. However, environmental factors such as climate, soil’s basic physical and chemical properties, agricultural infrastructure, and production conditions vary across regions, leading to different responses of wheat varieties to water and fertilizer regulation (Sun et al., 2024; Dupont and Altenbach, 2003). Therefore, it remains crucial to thoroughly explore the production benefits of high-yield wheat variety quality and yield under various water and fertilizer management practices. Based on the integrated drip irrigation system of water and fertilizer in the Huang-Huai-Hai area, this experiment investigated deeply the irrigation model for synergistic improvement of wheat quality and yield with supplementary irrigation of water-fertilizer in different key growth periods of wheat by the analysis of soil moisture, aims to provide theoretical support for establishing a wheat cultivation technology system that promotes water and fertilizer savings, high yield, efficiency, and superior quality in the Huang-Huai-Hai area.

## 2 Materials and methods

### 2.1 Experimental site description

**Figure 1:**
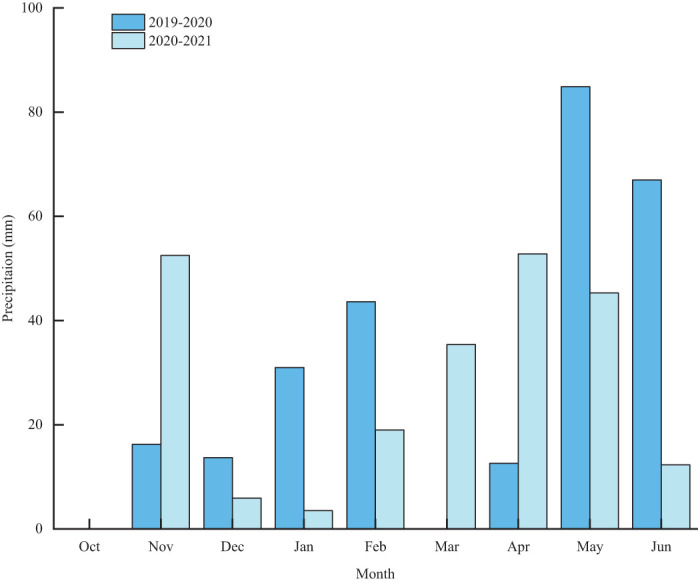
Effective precipitation during winter wheat growth period from 2019 to 2021.

The experiment was conducted over two growing seasons at the Jiaozhou Modern Agriculture Demonstration Park (35° 53’ N, 119° 58’ E) of Qingdao Agricultural University, spanning from 2019 to 2021. Prior to the cultivation of winter wheat, summer maize was grown during the 2019-2020 and 2020-2021 seasons. The region features a subhumid monsoon climate and the predominant soil type is lime concretion black soil. Details regarding the soil’s organic matter content, pH value, available phosphorus, available potassium, and alkali-hydrolyzed nitrogen in the top 20 cm of soil prior to seeding are detailed in **Table 1**. Monthly precipitation data for the period is presented in **Figure 1**.

**Table 1:** Basic physical and chemical properties of 0-20 cm soil layer of experiment field before sowing.

Growing season	Organic matter (g/kg)	Soil pH	Hydrolysable N (mg/kg)	Available P (mg/kg)	Available K (mg/kg)
2019-2020	17.33	7.83	125.40	17.13	135.02
2020-2021	17.12	7.62	118.31	16.84	133.03

### 2.2 Experimental design and crop management

The winter wheat variety “Ji Mai 22” was tested over two growing seasons. Two nitrogen application rates under drip irrigation were established: 180 kg/ha (N1) and 210 kg/ha (N2). The irrigation and fertilization were timed for several growth stages: jointing, anthesis (S2), jointing through filling (S3), and jointing through booting and filling (S4). Two soil layer depths were considered: 0-10 cm (M1) and 0-20 cm (M2). Under N1 and N2, six drip irrigation regimes were configured: S3M1, S3M2, S4M1, S4M2. A traditional border irrigation method served as the control (CK), which involved nitrogen application and irrigation at jointing and anthesis stages, resulting in a total of 13 treatments detailed in **Table 2**. Under drip irrigation, the average relative water content of the target soil layer was maintained at 100%. Water application was quantified using a water meter, with volumes detailed in **Table 3** for different stages.

**Table 2:** Experiments design.

Treatment	Soil layer depth (cm)	Nitrogen fertilization (kg/ha)			
		Jointing	Booting	Anthesis	Filling
CK	—	150			
N1S4M2	20	22.5	22.5	22.5	22.5
N1S4M1	10	22.5	22.5	22.5	22.5
N1S3M2	20	30		30	30
N1S3M1	10	30		30	30
N1S2M2	20	45		45	
N1S2M1	10	45		45	
N2S4M2	20	30	30	30	30
N2S4M1	10	30	30	30	30
N2S3M2	20	40		40	40
N2S3M1	10	40		40	40
N2S2M2	20	60		60	
N2S2M1	10	60		60	

CK, control; Nx (x = 1, 2), N1and N2, nitrogen application 180 kg/ha and 210kg/ha respectively; Sx (x = 2, 3, 4), drip irrigation frequency, 2, 3, 4 times respectively; Mx (x = 1, 2): soil layer 0–10 cm, 0–20 cm respectively; I, Irrigation frequency; N, Nitrogen application rate; S, soil layer depth.

**Table 3:** Irrigation volume at different stages in winter wheat.

Treatment	Jointing			Booting		Anthesis			Filling	
	N1	N2	N3	N1	N2	N1	N2	N3	N1	N2
2019-2020	10 April			17 April		6 May			16 May	
CK			98.7					66.6		
S4M2	64.4	63.8		59.9	61.5	23.1	24.8		39.9	39.3
S4M1	34.3	34.8		32.2	33.3	12.9	13.3		19.7	20.7
S3M2	68.7	66.9				27.1	28.4		42.3	39.5
S3M1	36.0	37				15.1	15		21.5	23.4
S2M2	73.5	72.3				30.5	31.2			
S2M1	38.5	39.2				16.6	16.9			
2020-2021	13 April			19 April		8 May			17 May	
CK			90.5					67.4		
S4M2	56.8	56.5		52.9	54.4	20.4	21.9		35.2	34.8
S4M1	27.3	28.1		25.7	26.9	10.2	10.7		15.7	16.7
S3M2	54.8	55.8				21.7	23.7		33.8	33
S3M1	27.6	28.8				11.6	11.6		16.4	18.2
S2M2	63.8	64.5				26.4	27.8			
S2M1	30.9	32.0				13.4	13.8			

CK, control; Nx (x = 1, 2), N1and N2, nitrogen application 180 kg/ha and 210kg/ha respectively; Sx (x = 2, 3, 4), drip irrigation frequency, 2, 3, 4 times respectively; Mx (x = 1, 2): soil layer 0–10 cm, 0–20 cm respectively; I, Irrigation frequency; N, Nitrogen application rate; S, soil layer depth.

**Figure 2:**
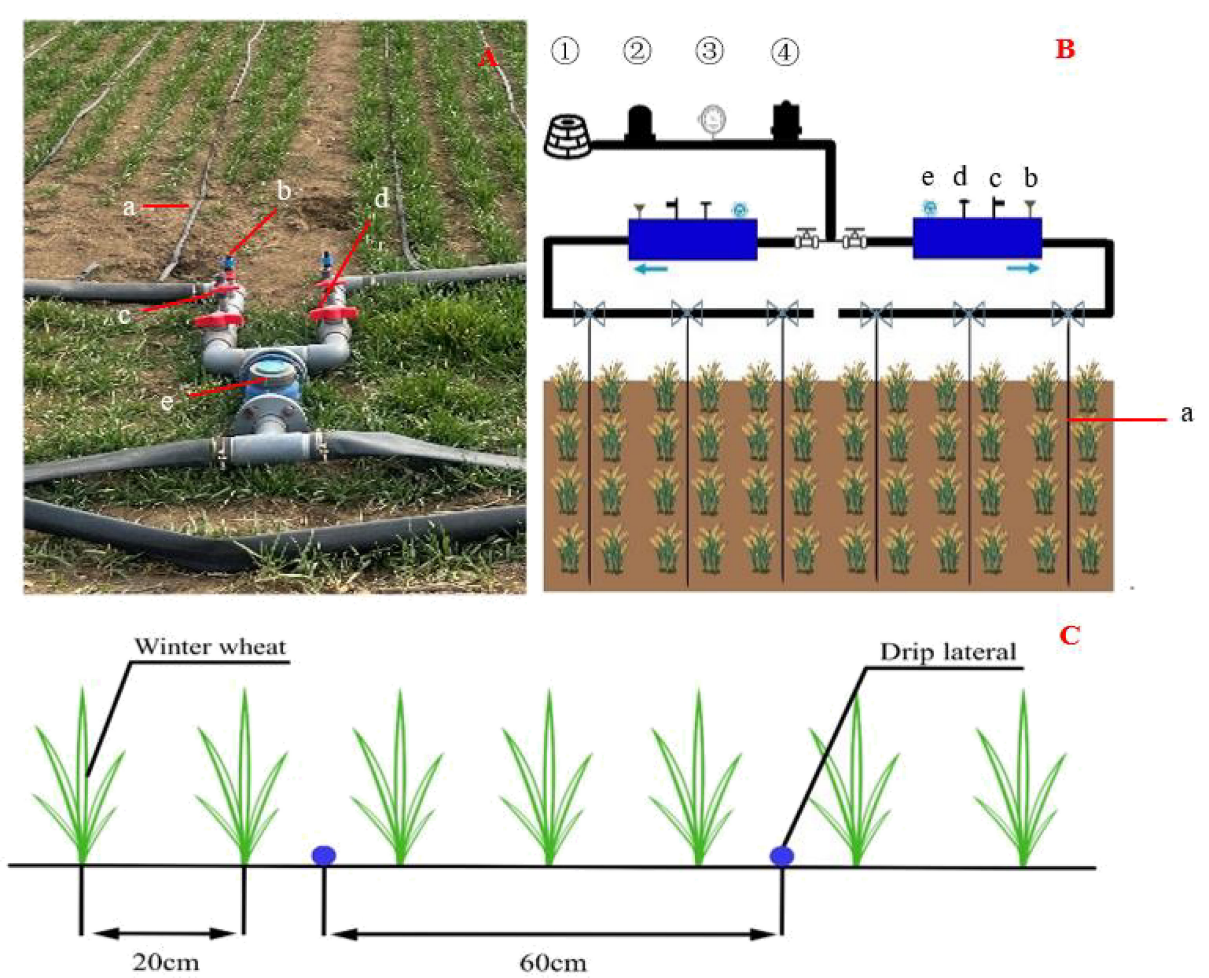
Laying of drip irrigation belts in the field. **(A)**, the actual situation of field drip irrigation system laying; **(B)**, the schematic diagram of drip irrigation belt laying; **(C)**, the parameters of drip irrigation belt laying; a, drip irrigation belt, b, fertilizer inlet, c, exhaust port, d, cut-off valve, e, water meter in **(A, B)**; ①, well, ②, pump,③, pressure gauge, ④, water filter in **(B)**.

The experimental field received compound fertilizer (N: P: K= 15:15:15) at the base rates of pure nitrogen, K_2_O, and P_2_O_5_ at 90 kg/ha each, supplemented by equal amounts of urea (46% nitrogen) mixed with irrigation water. The plots, measuring 2.4 m in width, 20 cm row spacing, and extending 60 m in length, were replicated thrice. Drip irrigation tapes were installed in a “one pipe, three rows” configuration with 60 cm between rows to ensure uniform water distribution (**Figure 2**). Corn straw was mulched back into the field before sowing, which followed tilling and rotating the soil. Sowing occurred on October 14, 2019, and October 10, 2020, with seeding rates of 150 kg/ha and 200 seeds per square meter, respectively. Harvesting was completed on June 16, 2020, and June 19, 2021. All other management practices aligned with those used in high-yield agriculture.

### 2.3 GY and yield components

At the maturity stage of winter wheat, the number of spikes, grains per spike, and the 1000-grain weight were assessed. To measure the yield, a random sample of 12 m² was harvested from each plot. The water content of the grain was determined to be 13.0%.

### 2.4 Soil moisture content

During each treatment period, soil moisture content in the top 20 cm was measured using the gravimetric method. The calculation for soil gravimetric moisture content (SGMC, %) was expressed as:


SGMC=SMC(g)/DSM(g)×100

Here, SMC and DSM are the soil moisture content and dry soil mass.

### 2.5 The amount of supplementary irrigation

Soil water storage (SWS, mm) and irrigation volume (IV, Guo et al., 2014) was calculated using the formula:


(2) $$\text{SWS }\left(\text{mm}\right)\;=\;0.1\times \text{ SBD }\left(\text{g}/{\text{cm}}^{3}\right)\;\times \text{ ST }\left(\text{cm}\right)\;\times \text{ SMWC}\left(\%\right)$$

(3) $$\text{IV }\left(\text{mm}\right)\;=\;10\times \text{ DIS}\left(\text{cm}\right)\;\times \text{ SBD }\left(\text{g}/{\text{cm}}^{3}\right)\;\times \;\left(\text{TWC }-\text{ SWCBI}\right)$$

Where SBD is soil bulk density, ST is soil thickness, SMWC is soil mass water content, DIS is depth of irrigated soil, TWC is target water content, SWCBI is soil water content before irrigation. IUE was calculated as:


(4) IUE (kg/(ha·mm)) = GY (kg/ha) / IV (mm)

### 2.6 Plant partial factor productivity of nitrogen fertilizer

PFPN was calculated as:


(5)  PFPN (kg/kg) = GY (kg/ha) / NFAR(kg/ha)

Here, NFAR is nitrogen fertilizer application amount.

### 2.7 Determination of wheat quality characters and pasting properties

Grain bulk density was measured using the SC6000TR (SC6000TR; Next Instruments, Australia), and grain hardness was assessed with the GWJ-2 hardness tester (GWJ-2; Hangzhou Dacheng Optoelectronic Instrument, China). Wheat grains were crushed using the N9548R mill (N9548R; HODER, China). Soluble sugar content in the wheat grains was quantified by the anthrone colorimetric method (Abid et al., 2018). The total nitrogen content of the wheat grain was analyzed using the Kjeldahl method, applying a grain protein conversion factor of 5.7. Amylose and amylopectin contents were determined via the dual-wavelength method; their combined total represents the overall starch content. Water absorption, dough formation time, and dough stability time were evaluated using the AACC54-21 standard method and a silty meter. The tensile index of flour was measured with a tension meter. The gluten index was determined using the Y07 automatic gluten cleaning instrument, gluten index tester, and Y09 gluten drying instrument (Y07, T08 and Y09; YUCEBAS, Turkey). Finally, the pasting viscosity characteristics of wheat grains were assessed using a Rapid Visco Analyzer (RVA-4; Newport Scientific, Australia).

### 2.8 Statistical analysis

Experimental results were processed, calculated, analyzed, and visualized using Microsoft Excel 2019 and Origin 2021. Statistical variance analysis (ANOVA) was performed using PASW Statistics 18.0, with significance thresholds set at p = 0.05. Visual representations were created in Origin Pro 2019.

## 3 Results

### 3.1 GY, PFPN and IUE

Field tests conducted from 2019 to 2021 (**Table 4**) revealed that different water and fertilizer schemes did not significantly affect the number of ears or grains per spike in wheat. However, there was a significant increase in 1000-grain weight compared to the CK under the 3 and 4 times irrigation treatments. Compared to CK, the biennial average yield of 3 and 4-irrigation treatments were significantly increased by 8.0% and 13.6%, respectively. PFPN decreased with the adding irrigation frequency and irrigation depth but IUE did the opposite. Compared with CK, PFPN of two-year average of 3 and 4 irrigations increased by 82.8% and 137.1%, and IUE increased by 37.8% and 31.6%, respectively. Overall, 1000-grain weight, GY, PFPN and IUE were significantly influenced by irrigation frequency and its interaction with nitrogen application and soil depth, although nitrogen application alone did not significantly alter these metrics.

**Table 4:** Effects of different treatments on grain yield, its components, PFPN and IUE.

Treatment	Spike number (×10^4^)	Kernels per spike	1000-grain weight (g)	GY (kg/ha)	PFPN (kg/kg)	IUE (kg/(ha·mm))
2019-2020						
CK	666.1a	29.9a	41.1b	6920.1b	41.9f	28.8d
N1S4M2	669.9a	30.4a	44.6a	7679.3a	41.0f	42.7a
N1S4M1	673.4a	30.1a	44.2a	7598.2a	76.7c	42.2a
N1S3M2	663.9a	30.2a	43.8a	7409.0a	53.6e	41.2a
N1S3M1	654.2a	30.0a	43.1a	7338.8a	101.1b	40.8a
N1S2M2	663.4a	29.8a	42.2b	7021.9ab	67.5d	39.0ab
N1S2M1	658.4a	29.4a	41.5b	6761.6b	122.7a	37.6b
N2S4M2	678.4a	31.1a	44.7a	7961.2a	42.0f	37.9b
N2S4M1	670.6a	31.2a	44.3a	7813.2a	76.5c	37.2b
N2S3M2	676.1a	30.4a	43.7a	7549.0a	56.0e	35.9bc
N2S3M1	668.0a	30.2a	43.5a	7355.3a	97.6b	35.0bc
N2S2M2	661.5a	30.4a	42.2ab	7067.0ab	68.3d	33.7c
N2S2M1	653.4a	29.5a	41.9b	6836.0b	121.9a	32.6c
2020-2021						
CK	676.5a	31.4a	45.8b	8227.7c	52.1f	34.3d
N1S4M2	699.8a	32.8a	48.1a	9338.6a	56.5f	51.9a
N1S4M1	705.3a	32.5a	47.8a	9276.4a	117.6c	51.5a
N1S3M2	692.6a	32.4a	47.6a	9006.6a	81.5e	50.0a
N1S3M1	681.2a	31.9a	47.4a	8707.9ab	156.6b	48.4ab
N1S2M2	689.3a	31.3a	46.4b	8420.7bc	93.4d	46.8b
N1S2M1	685.1a	31.2a	45.9b	8252.7c	186.3a	45.8b
N2S4M2	711.9a	33.2a	48.3a	9650.3a	57.6f	46.0b
N2S4M1	703.1a	33.2a	48.1a	9490.4a	115.2c	45.2b
N2S3M2	705.3a	32.1a	47.7a	9133.0a	81.2e	43.5bc
N2S3M1	698.1a	32.0a	47.6a	8977.7ab	153.2b	42.8bc
N2S2M2	698.1a	31.6a	46.2b	8480.2bc	91.9d	40.4c
N2S2M1	687.3a	31.6a	46.1b	8337.0c	182.0a	39.7c
ANOVA						
I	ns	ns	**	**	**	**
N	ns	ns	ns	ns	ns	ns
S	ns	ns	*	*	ns	*
I×N	ns	ns	*	*	*	*
I×S	ns	ns	*	*	*	*
N×S	ns	ns	ns	ns	ns	*
I×N×S	ns	ns	*	*	*	*

PFPN, partial factor productivity of nitrogen fertilizer; IUE, irrigation use efficiency. CK, control; Nx (x = 1, 2), N1and N2, nitrogen application 180 kg/ha and 210kg/ha respectively; Sx (x = 2, 3, 4), drip irrigation frequency, 2, 3, 4 times respectively; Mx (x = 1, 2): soil layer 0–10 cm, 0–20 cm respectively; I, Irrigation frequency; N, Nitrogen application rate; S, soil layer depth. I×N, I×S, N×S and I×N×S represent the interaction between irrigation frequency and nitrogen application rate, irrigation frequency and soil depth, nitrogen application rate and soil depth, and the interaction between irrigation frequency, nitrogen application rate and soil depth, respectively. ns, no significance. Different letters after numbers indicate significance at *P*<0.05. “*” and “**” after F-values represent significant difference at *P*<0.05 and *P*<0.01, respectively. Same as below.

### 3.2 Grain processing quality

Over the two-year wheat growing seasons, as detailed in **Table 5**, the general trend indicated that the GC and Zeleny Sedimentation Volume (ZSV) of wheat increased with the frequency of irrigation under two nitrogen application rates. The 3and 4 times irrigation treatments increased significantly the two-year averages of GC by 1.6% and 5.1% respectively compared to CK, with no difference in ZSV. According to the performance of DT, ST, water absorption (WAS) and gluten hardness (GH), the processing performance of S4M1, S3M2 and S3M1 is the most stable. The enzyme activity (EA), dough elongation (DE) and maximum resistance (Rmax) decreased with the increase of irrigation frequencies under the same nitrogen application rate and irrigation soil depth under drip irrigation conditions, although the 4-irrigations showed a decrease in some indexes, other drip irrigation treatments still work better compared with CK. Interaction effect analysis revealed that the grain processing quality was significantly influenced by the combined effects of irrigation frequency, nitrogen application gradient, and irrigation depth, whereas the responses to the individual factors of nitrogen gradient and supplementary irrigation depth were minimal.

**Table 5:** Effects of different treatments on processing quality of winter wheat.

Treatment	GC (%)	ZSV(mL)	DT (s)	ST (s)	WAS (%)	GH (%)	BW (g/L)	EA (cm^2^)	DE (mm)	Rmax (BU)
2019-2020										
CK	32.8a	35.8a	204a	331a	58.4a	53.3a	812.5ab	44.7a	155.3a	212.5a
N1S4M2	33.8a	36.8a	180ab	288bc	51.6b	50.9b	816.3a	39.5b	142.7b	175.3b
N1S4M1	32.2a	35.4a	192a	306ab	54.3ab	52.5ab	814.2ab	42.4ab	145.4b	183.5ab
N1S3M2	32.0a	35.9a	194a	330a	53.5ab	51.4ab	815.1ab	42.2ab	149.2ab	181.7ab
N1S3M1	31.4ab	34.6a	183ab	312ab	54.1ab	52.9ab	813.7ab	41.7ab	151.9ab	187.8ab
N1S2M2	30.3b	32.4b	192a	305b	56.3a	53.5a	811.6b	44.6a	154.1a	210.4a
N1S2M1	29.6b	31.6b	168b	282bc	59.2a	54.0a	810.3b	46.3a	156.5a	214.1a
N2S4M2	33.9a	36.8a	186a	286bc	52.1b	50.7b	817.4a	38.1b	144.6b	176.2b
N2S4M1	32.7a	37.1a	198a	306ab	54.3ab	52.5ab	814.6ab	42.5ab	146.3b	183.5ab
N2S3M2	32.9a	35.6a	204a	330a	53.8b	51.9ab	815.5a	43.1ab	150.1ab	182.2ab
N2S3M1	32.2a	35.4a	193a	318ab	55.4ab	53.8a	814.0ab	43.5ab	152.8a	187.3ab
N2S2M2	30.0b	33.3b	192a	297b	55.3ab	53.8a	811.9b	45.4a	155.1a	211.4a
N2S2M1	29.7b	32.3b	174b	276c	60.6a	54.1a	810.6b	47.2a	156.6a	215.7a
2020-2021										
CK	30.5b	35.3a	202a	328a	55.6a	52.2a	781.4ab	42.3a	151.4a	208.3a
N1S4M2	33.3a	36.2a	177b	280bc	50.3b	49.7b	784.1a	37.4b	138.7b	170.1b
N1S4M1	32.6a	34.1ab	195a	301ab	53.2ab	50.7ab	783.1ab	41.8ab	140.3b	178.2ab
N1S3M2	32.3a	34.7ab	191a	317a	52.9ab	50.5ab	783.9a	40.6ab	145.1ab	175.4ab
N1S3M1	32.0a	33.9ab	180a	297ab	52.ab	51.1ab	782.6ab	39.2ab	148.7ab	183.3ab
N1S2M2	27.6c	32.7b	186a	283b	53.6ab	51.7ab	780.6ab	42.2a	149.8ab	205.6a
N1S2M1	27.0c	30.3b	162b	270bc	56.4a	52.2a	777.3b	43.6a	152.2a	209.0a
N2S4M2	34.2a	37.1a	180ab	278bc	50.5b	50.1ab	786.2a	37.9b	139.5b	172.0b
N2S4M1	33.5a	36.8a	186a	299ab	52.7ab	50.7ab	783.5ab	38.6b	141.2b	179.2ab
N2S3M2	32.7ab	33.8ab	198a	312a	52.2ab	50.1ab	784.3ab	41.5ab	147.8ab	177.9ab
N2S3M1	31.8ab	33.2ab	194a	300ab	53.7ab	52.0a	781.7ab	41.9ab	149.7a	182.9ab
N2S2M2	28.3c	32.8b	192a	288b	54.3ab	52.4a	776.9b	42.3a	150.8a	206.4a
N2S2M1	27.4c	31.7b	168b	264c	57.8a	52.3a	775.6b	43.8a	153.3a	210.6a
ANOVA										
I	**	**	**	**	**	**	*	**	**	**
N	ns	ns	ns	ns	ns	ns	ns	ns	ns	ns
S	ns	ns	ns	ns	ns	ns	ns	ns	ns	ns
I×N	*	ns	ns	ns	ns	ns	ns	ns	ns	ns
I×S	*	ns	ns	ns	ns	ns	ns	ns	ns	ns
N×S	*	*	ns	*	ns	ns	ns	ns	ns	ns
I×N×S	**	*	*	**	*	*	*	*	*	*

GC, wet gluten content; ZSV, zeleny sedimentation value; DT, development time; ST, stable time; WAS, water absorption speed; GH, grain hardness; BW, Bulk weight; EA, Extension area; DE, dough extensibility; Rmax, Resistance at max. CK, control; Nx (x = 1, 2), N1and N2, nitrogen application 180 kg/ha and 210kg/ha respectively; Sx (x = 2, 3, 4), drip irrigation frequency, 2, 3, 4 times respectively; Mx (x = 1, 2): soil layer 0–10 cm, 0–20 cm respectively; I, Irrigation frequency; N, Nitrogen application rate; S, soil layer depth. I×N, I×S, N×S and I×N×S represent the interaction between irrigation frequency and nitrogen application rate, irrigation frequency and soil depth, nitrogen application rate and soil depth, and the interaction between irrigation frequency, nitrogen application rate and soil depth, respectively. ns, no significance. Different letters after numbers indicate significance at *P*<0.05. “ * “ and “ ** “ after F-values represent significant difference at *P*<0.05 and *P*<0.01, respectively.

### 3.3 Starch and protein dynamics

From 2019 to 2021, as reported in **Table 6**, the trends for amylose, amylopectin, and total starch content in wheat grains generally increased and then decreased as irrigation frequency intensified across each nitrogen gradient. Specifically, the amylose content and amylopectin content over the two-year average performed best in the 3rd irrigation treatments, significantly increased by 9.4% and 11.4% respectively compared to CK, even the total starch content increased by 9.8%. In terms of the amylose to amylopectin ratio, 3rd irrigation treatments performed relatively stable. Additionally, as irrigation frequency rose, both crude protein and soluble sugar content in drip-irrigated treatments generally decreased, yet remained significantly above CK levels. Over the two-year period, average increases in crude protein and soluble sugar content were 4.9% and 7.4%, 6.5% and 9.8%, and 9.3% and 11.7% respectively compared to CK. The starch, protein, and sugar content in wheat were significantly influenced by the interaction between irrigation frequency, nitrogen application rate, and irrigation depth.

**Table 6:** Effects of different treatments on nutritional quality of winter wheat grains.

Treatment	Amylose Content (%)	Amylopectin Content (%)	Total starch content (%)	Ratio of amylose to amylopectin (%)	Protein content (%)	Soluble sugar content (mg/g)
2019-2020						
CK	13.9b	41.7c	55.6bc	33.3a	14.5c	16.4c
N1S4M2	12.3c	42.4b	54.7c	29.0b	15.0b	17.6b
N1S4M1	13.2b	42.9b	56.1bc	30.8b	15.2ab	17.8b
N1S3M2	14.9a	45.8a	60.7a	32.5ab	15.2ab	18.2a
N1S3M1	15.2a	46.7a	61.9a	32.5ab	15.4a	18.4a
N1S2M2	14.1ab	42.3b	56.4b	33.3a	15.7a	18.5a
N1S2M1	14.4ab	43.2b	57.6ab	33.3a	15.8a	18.7a
N2S4M2	12.4c	42.8b	55.2bc	29.0b	15.1b	17.5b
N2S4M1	13.2b	43.0b	56.2bc	30.7b	15.2ab	17.6b
N2S3M2	15.1a	46.1a	61.2a	32.8ab	15.3ab	17.7ab
N2S3M1	15.6a	47.1a	62.7a	33.1a	15.5a	17.7ab
N2S2M2	14.3ab	43.0b	57.3ab	33.3a	15.7a	17.9ab
N2S2M1	14.8ab	43.5b	58.3ab	34.0a	16.0a	18.0ab
2020-2021						
CK	14.1b	44.6c	58.7b	31.6a	13.4c	16.5c
N1S4M2	12.4d	45.4bc	57.8b	27.3b	14.0b	17.9ab
N1S4M1	13.3c	45.9bc	59.2b	29.0ab	14.1ab	18.0ab
N1S3M2	15.2a	49.0a	64.2a	31.0a	14.2ab	18.5a
N1S3M1	15.4a	50.0a	65.4a	30.8ab	14.4a	18.6a
N1S2M2	14.2b	45.3bc	59.5b	31.4a	14.6a	18.7a
N1S2M1	14.5b	46.2b	60.7b	31.4a	14.7a	19.1a
N2S4M2	12.5d	45.8bc	58.3b	27.3b	14.2b	17.4b
N2S4M1	13.4c	46.0b	59.4b	29.1ab	14.3ab	17.5b
N2S3M2	15.2a	49.3a	64.5a	30.8ab	14.4ab	17.6ab
N2S3M1	15.9a	50.4a	66.3a	31.6a	14.5a	17.9ab
N2S2M2	14.5b	46.0b	60.5b	31.5a	14.7a	18.0ab
N2S2M1	15.1a	46.5b	61.6b	32.4a	14.8a	18.2ab
ANOVA						
I	**	**	**	**	ns	**
N	ns	ns	ns	ns	ns	ns
S	*	ns	ns	ns	ns	ns
I×N	*	**	**	*	*	*
I×S	*	*	**	*	*	*
N×S	ns	*	*	ns	ns	ns
I×N×S	*	**	*	*	*	*

CK, control; Nx (x = 1, 2), N1and N2, nitrogen application 180 kg/ha and 210kg/ha respectively; Sx (x = 2, 3, 4), drip irrigation frequency, 2, 3, 4 times respectively; Mx (x = 1, 2): soil layer 0–10 cm, 0–20 cm respectively; I, Irrigation frequency; N, Nitrogen application rate; S, soil layer depth. I×N, I×S, N×S and I×N×S represent the interaction between irrigation frequency and nitrogen application rate, irrigation frequency and soil depth, nitrogen application rate and soil depth, and the interaction between irrigation frequency, nitrogen application rate and soil depth, respectively. ns, no significance. Different letters after numbers indicate significance at *P*<0.05. “ * “ and “ ** “ after F-values represent significant difference at *P*<0.05 and *P*<0.01, respectively.

### 3.4 Gelatinization characteristics

The **Table 7** illustrates that the peak viscosity (PKV) and trough viscosity (TV) generally decreased with increasing irrigation frequency. Specifically, the S3M1 treatment remained stable and showed no significant difference compared to the second irrigation treatment. In terms of BDV, the best performance was observed in the N1S4M2 configuration during the 2 times irrigation. For CPV and CSV, the trend showed a decrease from the 2 to the 4 times irrigation treatments. PeT decreased as irrigation frequency increased, with the 2 times irrigation displaying the highest values, 4 times lowest. In two years, the PaT of 2 and 3 times treatments was always significantly differences with CK. Multifactorial interaction analysis indicated that the gelatinization characteristics of wheat grains were primarily influenced by irrigation frequency and its interaction with soil depth and nitrogen application. The effects of soil depth and nitrogen application as singular factors or in combination were not significant.

**Table 7:** Effects of different treatments on gelatinization characteristics of winter wheat grains.

Treatment	PKV (RVU)	TV (RVU)	BDV (RVU)	CPV (RVU)	CSV (RVU)	PeT (min)	PaT (°C)
2019-2020							
CK	2687.1b	1788.2b	898.9ab	3112.3a	1324.1a	6.0ab	85.8b
N1S4M2	2325.5c	1568.7c	756.8c	2634.9c	1066.2b	5.7b	85.0b
N1S4M1	2431.3bc	1614.7bc	816.6b	2801.4b	1186.7ab	5.9ab	86.4ab
N1S3M2	2501.4bc	1685.9bc	815.5b	2959.5ab	1273.6ab	6.0ab	87.1a
N1S3M1	2635.9ab	1747.2ab	888.7ab	3092.9a	1345.7a	6.1ab	87.6a
N1S2M2	2736.8a	1878.3a	858.5ab	3064.7ab	1186.4ab	6.3a	88.1a
N1S2M1	2894.3a	1932.4a	961.9a	3140.6a	1208.2ab	6.6a	88.7a
N2S4M2	2445.7c	1613.1c	832.6ab	2674.0c	1060.9b	5.7b	85.0b
N2S4M1	2644.8ab	1688.5c	956.3a	2827.6b	1139.1ab	5.9ab	86.4ab
N2S3M2	2710.3ab	1777.8b	932.5ab	2936.4ab	1158.6ab	6.0ab	87.1a
N2S3M1	2788.9a	1845.9ab	943.0a	2987.5ab	1141.6ab	6.1ab	87.6a
N2S2M2	2872.8a	1911.7a	961.1a	3110.2a	1198.5ab	6.3a	88.1a
N2S2M1	2979.2a	2006.2a	973.0a	3239.1a	1232.9ab	6.6a	88.7a
2020-2021							
CK	2540.7b	1723.2ab	817.5ab	3070.5a	1347.3a	5.8b	84.3b
N1S4M2	2205.3c	1521.6c	683.7c	2566.2c	1044.6b	5.7b	84.1b
N1S4M1	2324.7bc	1587.8c	736.9bc	2686.9c	1099.1ab	5.7b	84.5b
N1S3M2	2402.5bc	1617.3bc	785.2b	2822.4ab	1205.1ab	6.0ab	85.0ab
N1S3M1	2573.2ab	1694.0bc	879.2a	2921.5ab	1227.5ab	6.0ab	85.5ab
N1S2M2	2607.0a	1795.6a	811.4ab	2994.3ab	1198.7ab	6.1a	86.0a
N1S2M1	2757.0a	1872.4a	884.6a	3033.5a	1161.1ab	6.3a	86.5a
N2S4M2	2329.7c	1576.0c	753.7b	2641.5c	1065.5b	5.7b	84.0b
N2S4M1	2433.1bc	1673.6bc	759.5b	2710.3bc	1036.7b	5.8b	84.5b
N2S3M2	2509.2ab	1786.3a	722.9bc	2804.6ab	1018.3b	6.1a	85.2ab
N2S3M1	2577.6ab	1821.2a	756.4b	2905.9ab	1084.7ab	6.1a	86.6a
N2S2M2	2701.5a	1860.5a	841.0ab	3022.0a	1161.5ab	6.2a	87.1a
N2S2M1	2824.9a	1917.0a	907.9a	3164.8a	1247.8ab	6.3a	87.7a
ANOVA							
I	*	*	*	*	*	*	*
N	ns	ns	ns	ns	ns	ns	ns
S	*	ns	*	ns	ns	*	*
I×N	*	*	*	ns	ns	ns	ns
I×S	ns	ns	ns	ns	ns	ns	ns
N×S	ns	ns	ns	ns	ns	ns	ns
I×N×S	*	*	*	*	*	*	*

PKV, Peak viscosity; TV, Trough viscosity; BDV, Break down; CPV, Final viscosity; CSV, consistence; PeT, Peak time; PaT, Pasting temperature.CK, control; Nx (x = 1, 2), N1and N2, nitrogen application 180 kg/ha and 210kg/ha respectively; Sx (x = 2, 3, 4), drip irrigation frequency, 2, 3, 4 times respectively; Mx (x = 1, 2): soil layer 0–10 cm, 0–20 cm respectively; I, Irrigation frequency; N, Nitrogen application rate; S, soil layer depth. I×N, I×S, N×S and I×N×S represent the interaction between irrigation frequency and nitrogen application rate, irrigation frequency and soil depth, nitrogen application rate and soil depth, and the interaction between irrigation frequency, nitrogen application rate and soil depth, respectively. “ns”, no significance. Different letters after numbers indicate significance at P<0.05. “ * “after F-values represent significant difference at P<0.05.

### 3.5 Correlation between grain processing quality and gelatinization characteristics

**Figure 3:**
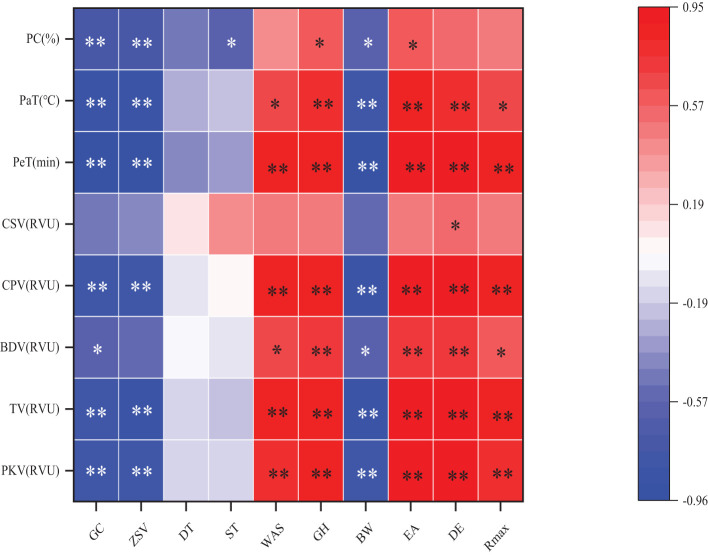
Correlation analysis between grain processing quality and gelatinization characteristics. PKV, Peak viscosity; TV, Trough viscosity; BDV, Break down; CPV, Final viscosity; CSV, consistence; PeT, Peak time; PaT, Pasting temperature, PC, protein content; GC, wet gluten content; ZSV, zeleny sedimentation value; DT, development time; ST, stable time; WAS, water absorption speed; GH, grain hardness; BW, Bulk weight; EA, Extension area; DE, dough extensibility; Rmax, Resistance at max. “ * “ and “ ** “ after F-values represent significant difference at *P*<0.05 and *P*<0.01, respectively.

According to a two-year correlation analysis (**Figure 3**), grain processing quality, gelatinization indices, and protein content demonstrated certain correlations. Notably, wet gluten content, sedimentation value, bulk density, gelatinization temperature, peak time, final viscosity, valley viscosity, and peak viscosity were all significantly negatively correlated. Similarly, disintegration value showed significant negative correlations with wet gluten content, stable time, and crude protein content. Conversely, processing attributes such as water absorption, hardness, tensile area, ductility, and maximum resistance positively correlated with peak time, final viscosity, valley viscosity, and peak viscosity. Additionally, hardness, tensile area, ductility, gelatinization temperature, and disintegration value also exhibited positive correlations. Significant positive correlations were observed between water absorption and both gelatinization temperature and disintegration value; hardness and protein content; tensile area and protein content; ductility and recovery value; and maximum resistance with both gelatinization temperature and disintegration value. No significant correlations were found among other quality indices.

## 4 Discussion

Irrigation significantly impacts grain yield and quality during the critical growth period. The synergistic application of water and fertilizer across various growth stages enhances their interaction, promoting effective nutrient absorption and contributing to yield formation (Wang et al., 2021). With a nitrogen application rate of 240 kg/ha and irrigation 120 mm from the jointing to filling, both yield and nutrient use efficiency are notably improved, and implementing integrated drip irrigation with 300 kg/ha of nitrogen didn’t result in high yields furthermore (Si et al., 2020). Following a fertilizer application ratio of basal fertilizer: dressing fertilizer = 50%:50% with 240 kg/ha and the relative water content in the 0∼40 cm soil layer was supplemented to 70% in the filled pore space at the jointing and flowering stages increased yield by 5.29∼15.34% (Zhang et al., 2021). Under the total nitrogen application rate of 210 kg/ha, three nitrogen base to topdressing ratios, 1000-grain weight and total protein content were significantly increased by 7.6% and 48.6% (Yao et al., 2023). This study observed no significant changes in panicle and grain numbers per panicle with 3 and 4 drip irrigations compared to CK, but saw substantial increases in thousand-grain weight and average yields over two years by 10.8%, with PFPN gains of 59.9%, IUE was also risen by 38.5% comprehensively, yet no substantial variance was found between the two irrigation levels. Multi-factor analysis indicated that thousand-grain weight, GY, PFPN and IUE were significantly influenced by irrigation frequency, as well as by its interactions with nitrogen application rate and soil depth. These results demonstrate that moderately increasing the irrigation frequency of post-anthesis was conducive to maintaining positive growth conditions of plants, combined with balanced fertilizer applications, enhance wheat’s growth characteristics, optimize environmental conditions for late growth phases, improving the utilization of water and fertilizer by plants, promoting the accumulation of biomass and eventually transferring it to seeds, and finally increasing the quality of seeds, achieving higher yields.

The strategic application of water and fertilizer significantly influences wheat grain yield and quality. Across two nitrogen application gradients, GC and ZSV increased with rising irrigation frequency, with the GC showing a notable 1.6% increase over two years compared to the CK under 3 irrigations treatments. Under consistent nitrogen gradient and irrigation depth, DT and ST initially rose then fell with increasing irrigation frequency, while WAS and GH gradually decreased, but BW increased. Besides, while two irrigation treatments and some indicators in the S4M2 treatment showed declines, other drip irrigation approaches displayed no significant differences from CK. Because the effective water and nitrogen management optimizes the nutrient supply structure, minimizes unproductive and excessive growth, and enhances grain quality by facilitating the transfer and storage of assimilates (Wang et al., 2023). Water scarcity during critical growth periods inhibit the transport of assimilates from vegetative plant parts to ear, especially during the filling, which was also influenced by genetic factors, these seriously affecting wheat settlement value, wet gluten content, and dough stability time (Wang et al., 2005; Tian et al., 2007). Therefore, uniform irrigation during the booting, jointing, anthesis, and filling stages substantially increases wheat bulk density, flour yield, gluten content, and settlement value, and improves several flour tensile properties including stability time, paste temperature, peak time, stretch area, elongation, and grain protein content (Xu et al., 2024). Obviously, the physiological process of dry matter transfer also affects starch content, because amylose and amylopectin, constituting over 65% of the grain’s dry matter, significantly influence processing quality. Research during the wheat filling stage reveals that water and fertilizer treatments markedly impact the soluble sugar and starch content, often showing inverse trends.

Over-irrigation in late growth stages dilutes grain quality (Lv et al., 2021), but total starch contents initially increase with irrigation frequency subsequently decrease, counteracting the negative effects of excessive watering, which can delay ripening and disperse energy needed for reproductive development, reducing starch transfer to grains (Xin et al., 2023; Scofield et al., 2009). Meanwhile, soluble sugars which primarily stored as water-soluble carbohydrates distributed in sucrose form and were crucial for starch synthesis, significantly impacted starch accumulation (Zi et al., 2018). The two-year averages for amylose and amylopectin in three-irrigation treatments rose by 9.4% and 11.4% respectively compared to CK, with total starch increasing by 9.8%. Protein and soluble sugar contents in drip irrigation treatments also showed marked increases over CK, enhancing grain nutrition quality. The above results showed that excessive irrigation, particularly in wet years, may not necessarily boost production and instead adversely affect IUE. Starch gelatinization characteristics—critical for starch application—were also variably affected by irrigation due to differences in starch granule structure among wheat varieties (Xiong et al., 2014). The PKV and TV of treatment S3M1, for example, diminish with increased irrigation. Overall performance trends show that lesser irrigation often outperforms more frequent watering, with significant performance declines noted in the fourth irrigation treatments over two years. These dynamics likely relate to annual precipitation variations, underscoring the influence of rainfall alongside irrigation on wheat quality and nutrient efficiency.

Comprehensive analysis of multifactorial interactions confirms that wheat grain quality is principally shaped by irrigation frequency and its interaction with soil depth and nitrogen levels, though these factors alone are less impactful. Correlation studies further validate the strong links between grain quality, gelatinization properties, and protein content. Given these insights, optimizing drip irrigation schemes is essential for maximizing yield, efficiency, and quality in dryland agriculture, particularly in the Huang-Huai-Hai region, aligning with national food security goals.

## 5 Conclusion

Analysis of the two-year averages, compared to CK, the sum of 3 and 4 irrigations treatments together significantly improved the GY, PFPN, and IUE respectively by 10.8%, 59.9% and 37.0%, with no significant differences between 3 and 4 irrigations. The GC, DT, ST, WAS, and GH of treatments S4M1, S3M2, and S3M1 outperformed others, amylose, amylopectin, and total starch contents in these three treatments significantly rose by 9.4%, 11.4%, and 9.8%. Meanwhile, 3 and 4 irrigations increased the crude protein and soluble sugar content by 4.9% and 7.4%, 6.5% and 9.8%, respectively, but the performance of 3irrigations in BDV, CPV, CSV, Pet and Pat were more effective than 4 irrigation treatments. In conclusion, 3 times irrigation generally yielded the best overall performance, aligning with the principles of water and fertilizer conservation, excellent yield, and high-quality production. The N1S3M1 treatment is recommended as an efficient management mode for high-yield production in the Huang-Huai-Hai area. Given the ample rainfall during the wheat growing season, N1S3M2 could be adopted in drier years to optimize resource use and crop outcomes.

## Data Availability

The original contributions presented in the study are included in the article/supplementary material. Further inquiries can be directed to the corresponding author.
